# Indirect Effects of Impoundment on Migrating Fish: Temperature Gradients in Fish Ladders Slow Dam Passage by Adult Chinook Salmon and Steelhead

**DOI:** 10.1371/journal.pone.0085586

**Published:** 2013-12-31

**Authors:** Christopher C. Caudill, Matthew L. Keefer, Tami S. Clabough, George P. Naughton, Brian J. Burke, Christopher A. Peery

**Affiliations:** 1 Department of Fish and Wildlife Sciences, University of Idaho, Moscow, Idaho, United States of America; 2 National Marine Fisheries Service, National Oceanic and Atmospheric Administration, Northwest Fisheries Science Center, Fish Ecology Division, Seattle, Washington, United States of America; 3 Idaho Fisheries Resource Office, U.S. Fish and Wildlife Service, Ahsahka, Idaho, United States of America; Ghent University, Belgium

## Abstract

Thermal layering in reservoirs upstream from hydroelectric dams can create temperature gradients in fishways used by upstream migrating adults. In the Snake River, Washington, federally-protected adult salmonids (*Oncorhynchus* spp.) often encounter relatively cool water in dam tailraces and lower ladder sections and warmer water in the upstream portions of ladders. Using radiotelemetry, we examined relationships between fish passage behavior and the temperature difference between the top and bottom of ladders (∆T) at four dams over four years. Some spring Chinook salmon (*O. tshawytscha*) experienced ∆T ≥ 0.5 °C. Many summer and fall Chinook salmon and summer steelhead (*O. mykiss*) experienced ∆T ≥ 1.0 °C, and some individuals encountered ΔT > 4.0°C. As ΔT increased, migrants were consistently more likely to move down fish ladders and exit into dam tailraces, resulting in upstream passage delays that ranged from hours to days. Fish body temperatures equilibrated to ladder temperatures and often exceeded 20°C, indicating potential negative physiological and fitness effects. Collectively, the results suggest that gradients in fishway water temperatures present a migration obstacle to many anadromous migrants. Unfavorable temperature gradients may be common at reservoir-fed fish passage facilities, especially those with seasonal thermal layering or stratification. Understanding and managing thermal heterogeneity at such sites may be important for ensuring efficient upstream passage and minimizing stress for migratory, temperature-sensitive species.

## Introduction

River impoundment has strong direct and indirect effects on river corridors used by migrating fishes. The most direct effect is blocked passage of migrants. However, fishways provide upstream routes at many dams, and a large body of research has focused on attracting fish to fishway openings and providing suitable hydraulic conditions to allow dam passage [1-4]. Dams additionally affect fish behavior and physiology in the migration corridor by altering water temperature [5-7], dissolved gas concentrations [8,9], and other physiochemical conditions both upstream and downstream from the dam [10]. Temperature alteration is of particular concern because temperature plays a central role in regulating fish physiology, behavior, and survival [11-13]. Relative to free-flowing systems, upstream-migrating fish in impounded systems may encounter much warmer or cooler thermal environments, increased thermal heterogeneity, and potential thermal barriers. 

**Figure 1:**
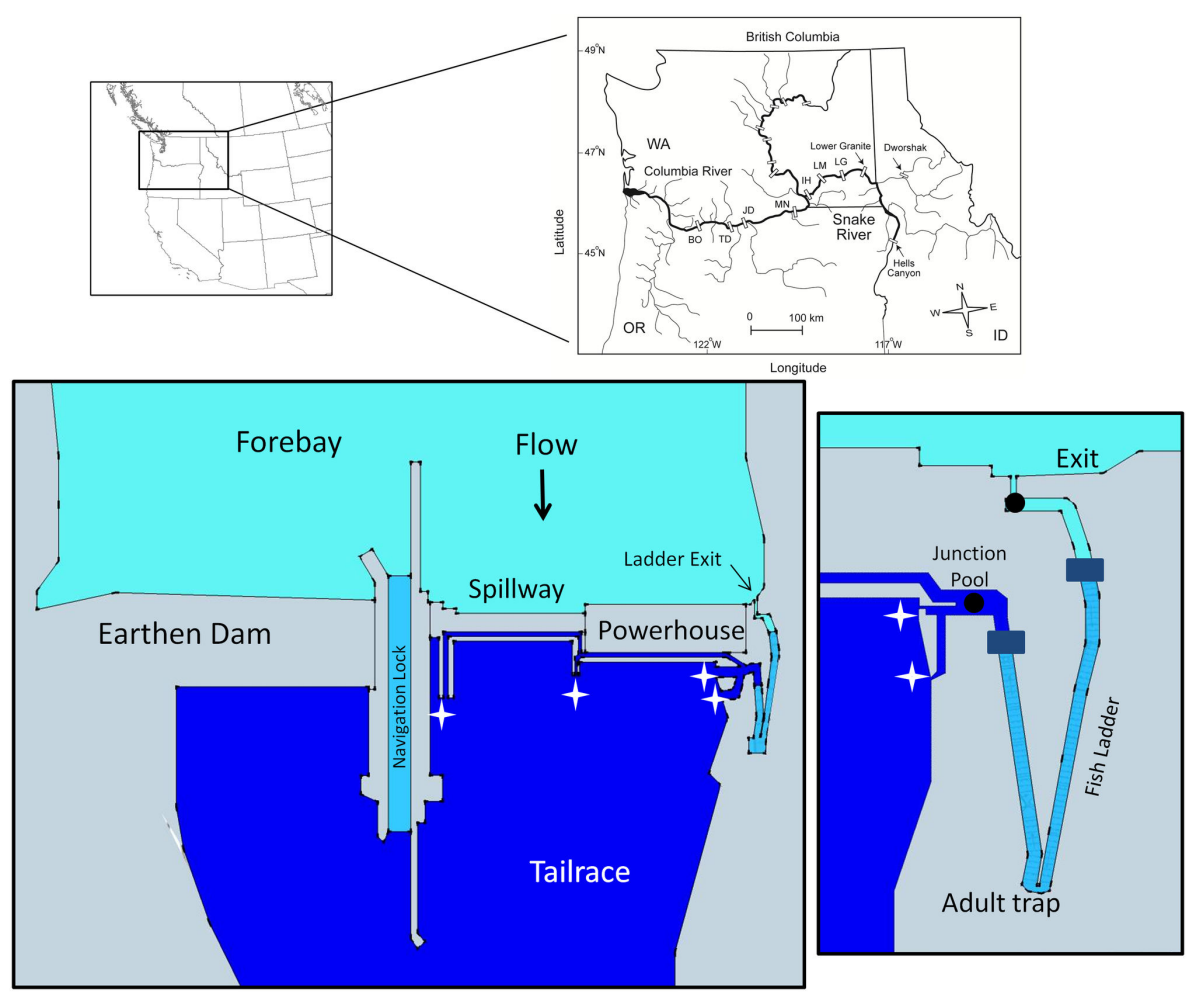
Map of the Snake River basin, showing locations of major dams and tributaries. Water temperatures and behaviors of radio-tagged adult Chinook salmon and steelhead were monitored at Ice Harbor (IH), Lower Monumental (LM), Little Goose (LG), and Lower Granite dams in 2000-2003. In all years, cold-water releases from Dworshak Dam on the Clearwater River affected thermal layering in Snake River reservoirs downstream. Large and small color insets are representations of Lower Granite Dam (975 m long, 30 m high), including fishway features typical of the Snake River dams. Colors indicate relatively warm forebay water, cool tailrace water, and intermediate fish ladder water. The fishway is composed of multiple tailrace entrances (white stars) where fish enter collection channels leading under the spillway and/or across the face of the dam. Channels merge at a junction pool and transition area at the base of the pool-and-weir ladder where tailrace water is pumped through diffusers to provide attraction flow at entrances (dark rectangles). The mid portion of the ladder is intermediate in temperature and is a mix of pumped tailrace water and forebay surface water. Dark circles indicate location of temperature loggers and radiotelemetry antennas used to estimate ΔT. BO = Bonneville Dam, TD = The Dalles Dam, JD = John Day Dam, MN = McNary Dam.

Reservoirs can increase water residence time and solar gain, accelerate spring warming, and delay fall cooling [14,15]. Importantly, reservoirs may also create vertical gradients in temperature caused by thermal layering or stratification in an otherwise well-mixed water column [16]. Thermal conditions in dam tailraces and reaches farther downstream can primarily be determined by reservoir surface water if releases are made over the dam spillway, or may be cooled by the release of hypolimnetic waters, as is the case for many hydroelectric dams drawing water through turbines [17]. Vertical temperature gradients in the reservoir result in thermal stratification that prevents mixing between layers or less distinct “thermal layering”, a precursor to full stratification. Such thermal gradients affect the temperature environment of fishways and fish ladders (the portions of most fishways that gain elevation) at dams. At most run-of-river Columbia and Snake River dams (Washington-Oregon), well-mixed water from dam tailraces is pumped into lower fishway segments, but upper sections of ladders are gravity-fed from seasonally warmer surface water in dam forebays (Figure 1 inset). 

Dams in the Snake River basin (Figure 1) have altered the overall thermal environment of the lower Snake River in several ways. Based on limited pre-dam data, it appears that mean annual temperatures and maximum temperatures have not dramatically changed, but the timing of spring warming has advanced and fall cooling has been delayed. The larger dam-related alteration of the summer thermal regime is produced by cold water released from the hypolimnion of Dworshak Reservoir on the Clearwater River, Idaho [16]. The Dworshak releases have occurred since 1991 in an effort to improve passage conditions for migrating juvenile and adult Pacific salmonids (*Oncorhynchus* spp) and have reduced summer mean temperatures. However, these releases have contributed to thermal layering in the reach between the Snake River-Clearwater River confluence and Lower Granite Dam [16]. In all four lower Snake River reservoirs, increased water residence times and solar heating cause additional thermal layering, especially in dam forebays. Prevailing upstream summer winds reinforce the layering by further slowing the movement of surface water masses. Such wind setup events can result in stratification and net transport of warm masses upstream over a deeper water mass moving downstream. The strongest stratification has been observed at Lower Granite Dam in summer, where forebay surface waters are often several degrees warmer than relatively well-mixed tailrace water. This pattern repeats at the dams farther downstream, but generally with smaller thermal gradients between the forebay and tailrace [18].

In this study, we examined the relationship between fish ladder temperature gradients at the four lower Snake River dams and passage behavior of several thousand radio-tagged adult Chinook salmon (*O. tshawytscha*) and steelhead (*O. mykiss*). Our primary objectives were to: (1) characterize water temperature differences (∆T) between base-of-ladder and top-of-ladder segments; (2) test for associations between ∆T and adult salmon and steelhead passage times through fish ladders; and (3) examine the relationship between internal fish body temperatures (estimated with combination radio-temperature logger tags) and fish ladder water temperatures. The collected data were sufficient to affirm our two principal hypotheses: (1) that fish passage time would increase as ∆T increased; and (2) that fish body temperatures would equilibrate to ambient temperatures prior to fish ladder exit into a dam forebay. 

## Methods

### Ethics Statement

The methods used to collect, radio tag, and monitor adult salmon and steelhead migration were approved by the University of Idaho Animal Care and Use Committee, have been detailed previously [25,26], and are summarized in the section “Radio Tagging and Telemetry Monitoring” below. Fish collection was permitted annually by NOAA Fisheries and the states of Washington and Oregon. 

### Study System

The Snake River is the largest Columbia River tributary, draining much of Idaho and parts of Oregon, Washington, Wyoming, Montana, Nevada, and Utah (Figure 1). Anadromous salmon and steelhead returning to the Snake River basin must pass four dams on the lower Columbia River and one to four lower Snake River dams (Figure 1). From downstream to upstream, the Snake River dams are Ice Harbor Dam (located ~538 river kilometers (rkm) from the Pacific Ocean), Lower Monumental Dam (rkm ~589), Little Goose Dam (rkm ~635), and Lower Granite Dam (rkm ~695). The head of Lower Granite reservoir fluctuates, but is generally near the confluence of the Snake and Clearwater rivers at rkm ~763. 

Below each of the four Snake River dams is a 1-2 km long tailrace with relatively turbulent flow caused by discharge from dam turbines and spillways. In tailraces, upstream migrants must distinguish between relatively low volume attraction flows leading to fishway entrances and the larger discharge from turbines and spillways. Once inside fishway entrances, fish first pass through collection channels that include attraction flow but no elevation gain, and then through junction pool and transition areas into fish ladders that gain ~30 m in elevation per dam. 

Mean hourly water temperature (°C) data were collected at multiple sites inside each fishway at the four Snake River dams [18]. Temperature data used in our analyses were from sites inside top-of-ladder exit areas (source water = forebay) and sites at the base of each ladder where fish collection channels transition to ladders (primary source water = tailrace). Ice Harbor and Lower Monumental dams have two fishways each, one adjacent to each shore; Little Goose and Lower Granite dams have single fishways on the south shore. 

### Study populations

Several Snake River salmon and steelhead populations are currently listed as threatened under the U.S. Endangered Species Act. These include spring-, summer-, and fall-run Chinook salmon and summer-run steelhead [19]. The three Chinook salmon runs are named after their adult migration timing, with spring-run fish passing through the lower Snake River from April to mid-June, summer-run fish passing in mid-June to mid-August, and fall-run fish passing in mid-August into late fall [20-22]. Adult summer steelhead pass through the lower Snake River from June through late fall, and some overwinter in the study reach before entering spawning tributaries the following spring [23]. Endangered Snake River sockeye salmon (*O. nerka*) are a summer-fall run [14,24], and reintroduced Snake River coho salmon (*O. kisutch*) are a fall run. The latter two species were not included in our telemetry study, but the temperature effects reported herein likely also apply. 

### Radio Tagging and Telemetry Monitoring

The methods used to collect, radio tag, and monitor adult salmon and steelhead have been detailed previously [25,26]. Briefly, from 2000-2003, adult fish were diverted from the Washington-shore fish ladder at Bonneville Dam (Columbia River rkm ~235 upstream from the Pacific Ocean and ~287 rkm downstream from the Snake River confluence) into a facility where they could be selected by species. Diverted fish were anesthetized, sexed, measured for fork length, and inspected for the presence of injuries and hatchery fin clips. Individual Chinook salmon were assigned to spring, summer, and fall runs using US Army Corps of Engineers criteria for passage date at Bonneville Dam [19]. We attempted to tag in proportion to the runs at Bonneville Dam, although temperature-related tagging restrictions occurred in most years. We could not distinguish Snake River fish from mid- and upper-Columbia River fish during tagging. Therefore, we used counts of adult migrants at Ice Harbor Dam to assess whether radio-tagged samples were proportional to the Snake River runs-at-large. 

All study fish were intragastrically tagged with a uniquely-coded radio transmitter (Lotek Wireless, Inc., Newmarket, Ontario) and released downstream from Bonneville Dam or in the Bonneville Dam forebay. Tagged fish were monitored at the four lower Snake River dams using aerial antennas in tailraces and underwater coaxial cable antenna arrays inside fishways. Antennas were located at fishway openings, inside collection channels, and at base-of-ladder and top-of-ladder sites. A subset of fish tagged in 2000 and 2002 received combination radio/data storage transmitters (RDST) that recorded depth every 5s and fish body temperature every 1 min. RDST transmitters were retrieved by diverting fish at the Lower Granite Dam fish trap on the Snake River [27], where transmitters were removed and fish were returned to the Snake River to continue migration. The Lower Granite adult trap is in the lower portion of the fish ladder, and consequently we did not examine patterns of fish body temperature at this location.

### Data analyses

We defined ∆T as the difference in mean hourly temperature between top-of-ladder and base-of-ladder sites at each fishway. Each radio-tagged fish was assigned a ∆T based on the date and time that it was first recorded at a base-of-ladder antenna at each dam. This was also the start time for fish passage time calculations. The ladder passage end time was defined as the time when a fish exited from the top of a fishway into a dam forebay. Elapsed time in between start and end times potentially included fish movement downstream into collection channels, exit into the tailrace, movement between fishways (Ice Harbor and Lower Monumental dams), fishway re-entries, and holding overnight in addition to passage up a ladder. We excluded time that fish spent after their first passage of a dam (i.e., if they fell back downstream over spillways or through turbines, and then re-attempted passage) because of the potential for learning or injury during fallback to affect passage behavior. We also censored a small number of fish that passed more than seven days after they were first detected at a dam, mostly steelhead that overwintered in the study area. 

We used generalized linear models (GLM; [28]) to assess whether fish passage time increased as ∆T increased at each dam (Hypothesis 1). Passage times were log_e_ transformed to improve normality and homogeneity of the error terms [29]. Model covariates were ∆T (°C), a quadratic effect of ladder passage start time (time of day), base-of-ladder water temperature at the start time, and migration year. Start time was included because many adult salmon and steelhead avoid hydraulically complex fishways at night [30] and those that enter fishways late in the day often hold overnight before passing [31]. Base-of-ladder temperature, a proxy for tailrace temperature, was included because adult salmon and steelhead migration speeds are often positively correlated with water temperature up to some threshold where activity is reduced [25,32,33]. We initially ran separate models for each run×dam×year combination (*n* = 60) to evaluate if main effects (∆T, base-of-ladder temperature, time of day) differed among years. Because patterns were generally consistent, we combined data from all years for each run×dam combination and included year as a covariate in model results. To illustrate summary results, we present model-averaged mean fish passage time estimates from annual GLMs at Lower Granite Dam using the 10^th^, 50^th^, and 90^th^ percentiles of encountered water temperatures for each run. 

The RDST data were used to test whether internal fish body temperatures equilibrated to water temperature in fish ladders (Hypothesis 2). Specifically, we examined individual fish telemetry and temperature histories to see if body temperatures matched water temperatures at each fish’s last detection in a fish ladder before entering a forebay. We restricted the RDST analysis to passage attempts where base-of-ladder and top-of-ladder records occurred on the same day because we were primarily interested in short-term fish body temperature changes as fish moved through thermal gradients within ladders. 

## Results

### Radio Tagging and the Runs-at-Large

In total, 3,416 spring Chinook salmon, 1,335 summer Chinook salmon, 3,842 fall Chinook salmon, and 4,199 summer steelhead were radio tagged and released near Bonneville Dam. Approximately 35% of spring Chinook salmon, 18% of summer Chinook salmon, 6% of fall Chinook salmon, and 44% of summer steelhead were subsequently detected at one or more Snake River dams and had enough telemetry detections to calculate ladder passage times (Table 1). On average, 87% (range = 54-100%) of the fish with ladder passage times were detected at times when ΔT data were available. 

**Table 1:** Number of radio-tagged Chinook salmon and steelhead used in the evaluation of ΔT effects on ladder passage times at Snake River dams in 2000-2003.

		**Chinook salmon**			**Steelhead**
**Dam**	**Year**	**Spring**	**Summer**	**Fall**	**Summer**
Ice Harbor	2000	189 (185)	44 (22)	45 (36)	459 (299)
	2001	427 (286)	71 (68)	93 (92)	437 (378)
	2002	305 (305)	67 (67)	73 (72)	600 (536)
	2003	255 (255)	59 (58)	29 (27)	193 (150)
	**Total**	**1,176 (1,031)**	**241 (215)**	**240 (227)**	**1,689 (1,363)**
Lower Monumental	2000	100 (85)	41 (41)	15 (15)	198 (198)
	2001	3 (3)	18 (18)	11 (11)	50 (50)
	2002	305 (305)	66 (66)	61 (61)	524 (522)
	2003	184 (180)	60 (60)	31 (31)	226 (226)
	**Total**	**592 (573)**	**185 (185)**	**118 (118)**	**998 (996)**
Little Goose	2000	201 (194)	41 (39)	33 (16)	458 (233)
	2001	430 (186)	71 (70)	81 (72)	452 (333)
	2002	305 (303)	67 (67)	62 (55)	621 (493)
	2003	262 (199)	60 (0)	30 (0)	269 (0)
	**Total**	**1,198 (882)**	**239 (176)**	**206 (143)**	**1,830 (1,059)**
Lower Granite	2000	84 (82)	41 (41)	23 (23)	208 (202)
	2001	247 (246)	69 (68)	71 (60)	309 (307)
	2002	289 (282)	66 (57)	57 (43)	458 (416)
	2003	29 (29)	12 (12)	n/a	11 (11)
	**Total**	**649 (639)**	**188 (178)**	**151 (126)**	**986 (936)**

Numbers in parentheses are those with both ladder passage times and calculable ΔT.

We compared the frequency distributions of radio-tagged adults to fish counts at Ice Harbor Dam for each year and run to assess how well tagged samples represented the Snake River runs-at-large. Spring Chinook salmon were slightly over-represented late in the run (June) and during the warmest temperatures experienced by this group in 2000 and 2001. Summer Chinook salmon were under-represented late in the runs (July-August) in all years, but particularly in 2000 and 2003. Fall Chinook salmon sample sizes were small but were generally proportional to fall Chinook salmon counts at the dams. Steelhead were under-represented early in each run (July-August) and were slightly over-represented late in the runs (mid-October to December). Most under sampling was caused by temperature-related tagging restrictions at Bonneville Dam, while steelhead over sampling was related to selection criteria at Bonneville Dam (Snake River stocks were targeted [26]). Collectively, the under and oversampling patterns suggest that summer Chinook salmon and steelhead runs-at-large likely experienced high ∆T more frequently than the radio-tagged samples, while spring Chinook salmon runs-at-large may have experienced high ∆T less frequently than radio-tagged samples.

### Water Temperature and ∆T

**Figure 2:**
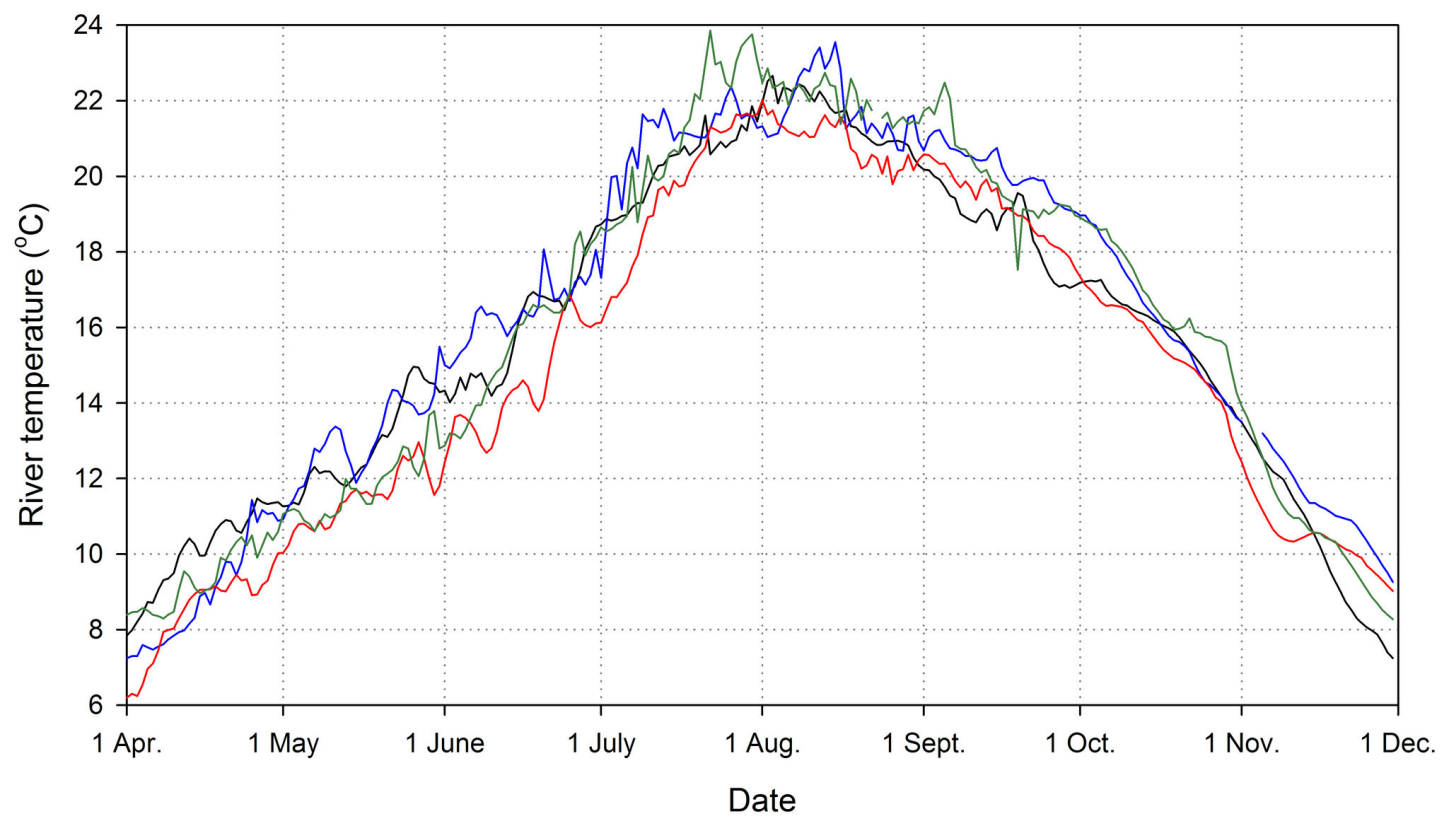
Mean daily water temperatures recorded at Ice Harbor Dam in 2000-2003. Temperatures were monitored by the U.S. Army Corps of Engineers in the dam forebay at a depth of 5 m; similar data were collected at Lower Monumental, Little Goose, and Lower Granite dams. Black line = 2000, blue line = 2001, red line = 2002, green line = 2003.

In each year, Snake River water temperature steadily increased from April until early to mid-August, and then decreased through the fall (Figure 2). Mean daily non-surface water temperatures at Ice Harbor Dam were ≥ 20 °C in each year from early to mid-July through early to mid-September. Annual maximum temperatures (from daily means) were 22.0-23.9 °C. On average, 2000 and 2002 were cooler than 2001 and 2003. Seasonal patterns and annual mean and maximum values were generally similar at the four dams.

**Figure 3:**
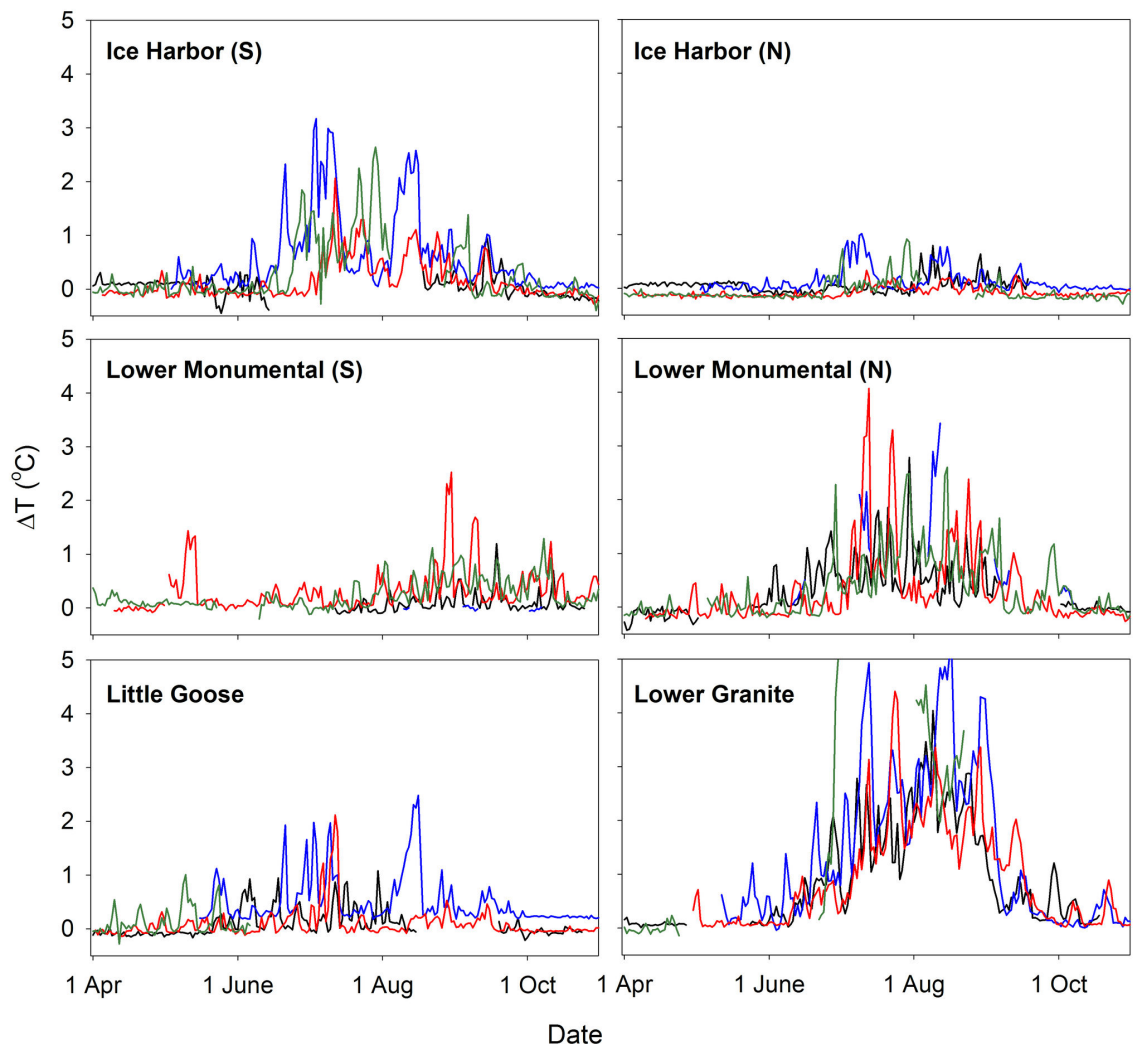
Mean daily ΔT estimates at six Snake River dam adult fish ladders in 2000-2003. ∆T was the water temperature difference between top-of-ladder and base-of-ladder monitoring sites inside fishways. S = South ladder, N = North ladder. Black line = 2000, blue line = 2001, red line = 2002, green line = 2003.

Ladder temperature differences (∆T) were calculable on 79% of all ladder×date combinations (*n* = 5,136 d) from 1 April to 31 October, 2000-2003 (Figure 3). The largest data gaps occurred at the Lower Monumental south ladder (2000, 2001), Lower Monumental north ladder (2001), Lower Granite ladder (2003), and Little Goose ladder (2003). The frequency and magnitude of ∆T generally increased with spring warming, was highest in summer, and declined with fall cooling. Observations of ∆T ≥ 2.0 °C were most frequent at the Lower Granite ladder, followed by the Lower Monumental north ladder, and the Ice Harbor south ladder. Maximum ∆T values exceeded 4°C on some days, mostly at Lower Granite Dam in summer. In contrast, ∆T infrequently exceeded 1.0 °C at the Ice Harbor north or Lower Monumental south ladders (Figure 3).

Diel fluctuations in ∆T were common, especially in summer and at the ladders with higher ∆T values (i.e., Lower Granite, Lower Monumental north, and Ice Harbor south). Typically, ∆T was lowest before sunrise and peaked in late afternoon or early evening. There was considerable day-to-day ∆T variability that was related to upstream dam operations (including Dworshak Dam on the Clearwater and Brownlee Dam on the upper Snake River, Figure 1), season, precipitation, cloud cover, and wind speed and direction (Pacific Northwest National Laboratory, Richland, Washington).

**Figure 4:**
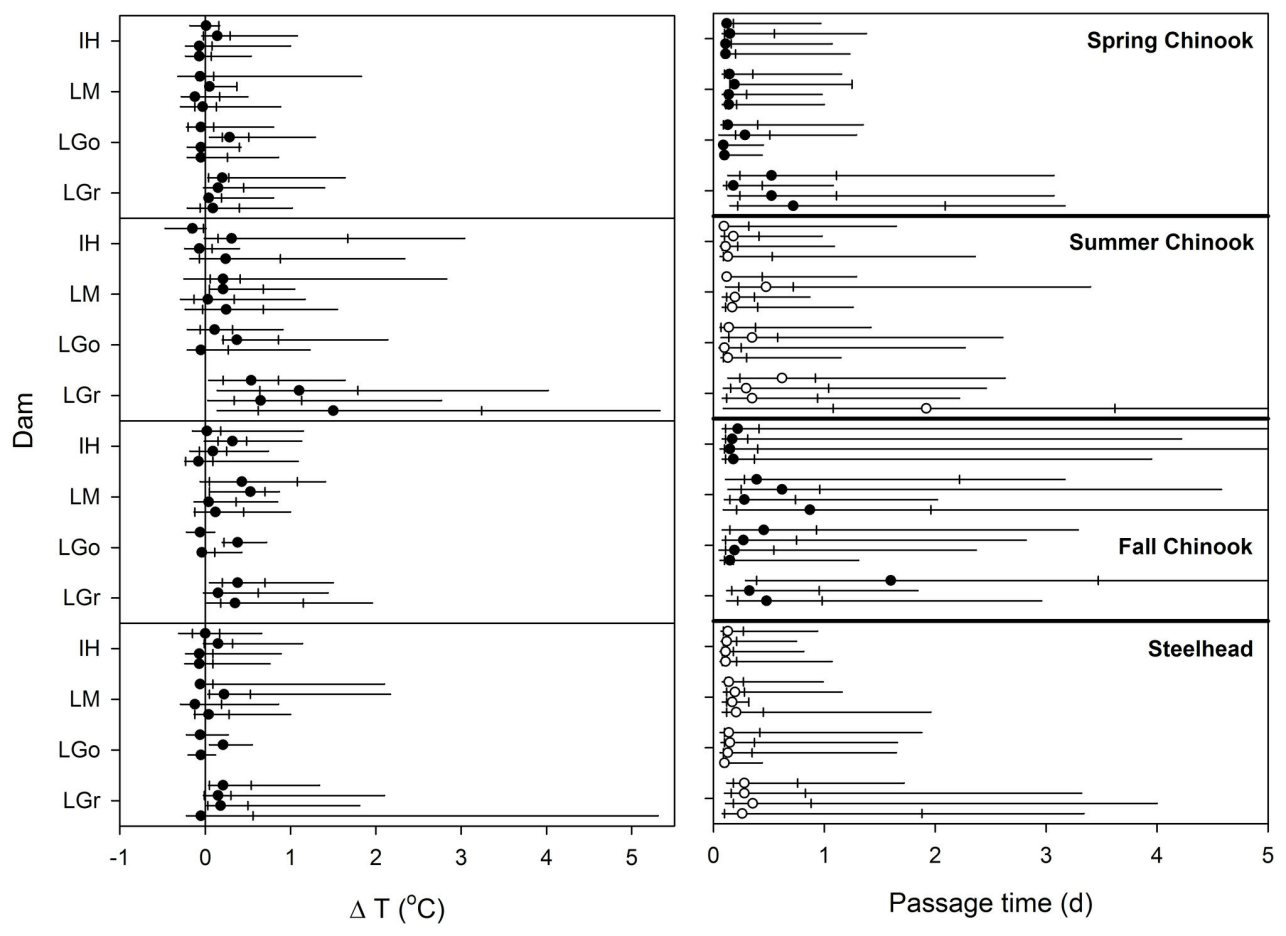
ΔT encountered by Chinook salmon and steelhead at fish ladders and associated fish passage times. Whisker plots show median, quartile, 5^th^ and 95^th^ percentiles. Years are sequential from 2000-2003, top to bottom, in each data cluster. Abbreviations as in Figure 1 except LGo = Little Goose Dam and LGr = Lower Granite Dam.

For individual fish with ladder passage times, ∆T values were collected for 60 run×dam×year combinations (Figure 4). Encountered ∆T varied widely among runs and among dams. Summer Chinook salmon and steelhead encountered the highest ∆T: 11 groups included fish that experienced ∆T > 2.0 °C and 3 experienced ∆T > 4.0 °C. Median run×dam×year ∆T values ranged from -0.15 to +1.50 °C. Medians were highest for summer Chinook salmon (*mean of* medians = +0.35 °C, *n* = 15 dam×year combinations) and fall Chinook salmon (+0.19 °C, *n* = 14). Means of medians were +0.03 for spring Chinook salmon (*n* = 16) and +0.05 for steelhead (*n* = 15). Averaged across dam×year combinations, 12% of radio-tagged spring Chinook salmon, 36% of summer Chinook salmon, 24% of fall Chinook salmon, and 15% of summer steelhead experienced ∆T ≥ 0.5°C. The run×dam combinations with the highest percentages of tagged fish encountering ∆T ≥ 0.5°C were summer Chinook salmon at Lower Granite (mean = 73%, *n* = 4 years), fall Chinook salmon at Lower Monumental (35%, 4 years), fall Chinook salmon at Lower Granite (34%, 4 years), summer Chinook salmon at Lower Monumental (27%, 4 years), and summer steelhead at Lower Monumental (25%, 4 years). 

### Relationship Between Fish Passage Time and ∆T

Most radio-tagged Chinook salmon and steelhead passed fish ladders on the day that they were first detected at a base-of-ladder antenna (Figure 4). Median ladder passage times in 63 run×dam×year estimates ranged from 2.2–46.1 h (mean of medians = 7.0 h). For all runs, the longest median times were at Lower Granite Dam. All ladder passage time distributions were right-skewed, with slower passage typically by fish that moved between fishways, exited fishways into a tailrace, or spent a night in a fishway or tailrace.

Higher rates of ∆T were associated with slower fish passage times in 13 of 16 run×dam combinations (see positive parameter estimates in Table 2), after accounting for year, time of day, and base-of-ladder water temperature. This association was significant (P < 0.05) for steelhead (all four dams), summer Chinook salmon (Ice Harbor and Lower Monumental dams), and fall Chinook salmon (Ice Harbor and Lower Granite dams), but not for spring Chinook salmon. Time of day was a significant covariate in 11 models, with longer passage times by fish that were first detected at base-of-ladder antennas late in the day or at night. Base-of-ladder water temperature was significant in 10 models, and 9 of 10 parameter estimates in these models were positive, indicating slower ladder passage as water temperature increased. The significant year effects in 8 of 16 models indicated that unmeasured covariates influencing fish passage time differed among years (e.g, dam operations, fish origin, maturation status, etc.).

**Table 2:** Parameter estimates from GLM models of fish ladder passage times in relation to ΔT.

			**Parameter estimate**			
**Dam**	**Run**	***n***	**Hour**	**Hour^2^**	**°C**	**ΔT**
Ice Harbor	Spring Chinook	1,026	-0.192^**^	0.010^**^	0.053^*^	-0.080
	Summer Chinook	215	-0.067	0.004	0.119^*^	0.355^**^
	Fall Chinook	218	-0.170^*^	0.009^**^	0.061	0.557^*^
	Steelhead	1,352	-0.239^**^	0.010^**^	-0.001	0.309^**^
Lower Monumental	Spring Chinook	572	-0.280^**^	0.012^**^	0.008	-0.039
	Summer Chinook	184	-0.064	0.003	0.125^**^	0.308^**^
	Fall Chinook	115	-0.118	0.007	-0.044	0.166
	Steelhead	987	-0.159^**^	0.007^**^	0.035^*^	0.124^*^
Little Goose	Spring Chinook	880	-0.040	0.002	0.045^*^	-0.122
	Summer Chinook	174	-0.082	0.003	0.179^**^	0.310
	Fall Chinook	142	-0.098	0.006	0.218^*^	0.792
	Steelhead	1,048	-0.114^**^	0.005^**^	0.066^**^	0.442^**^
Lower Granite	Spring Chinook	635	-0.130^**^	0.007^**^	0.022	0.047
	Summer Chinook	174	-0.077	0.003	0.159^**^	0.207^*^
	Fall Chinook	122	-0.141	0.007^*^	-0.112^*^	0.797^**^
	Steelhead	911	-0.115^**^	0.007^**^	-0.001	0.286^**^

Passage times were calculated for radio-tagged Chinook salmon and steelhead at Snake River dams in 2000-2003. Independent variables included migration year (not uniquely estimable), hour of first detection at a base-of-ladder antenna (quadratic effect), water temperature (°C) at the ladder base at the time of first detection, and ΔT (the °C temperature difference between top-of-ladder and base-of-ladder sites). Positive ΔT parameter estimates indicate slower fish passage as ΔT increases.

^*^ *P* ≤ 0.05; ^**^ *P* ≤ 0.005

**Figure 5:**
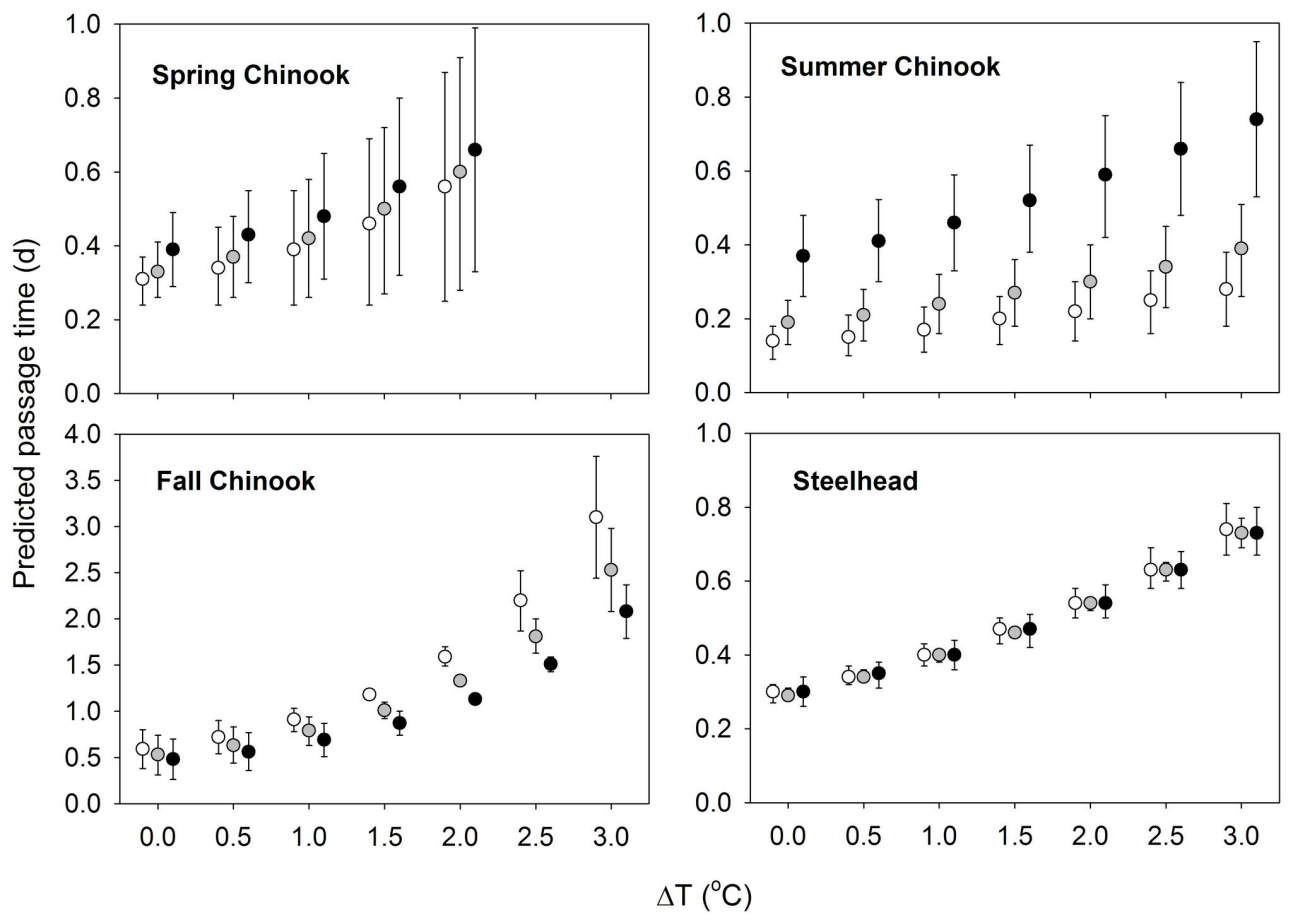
Predicted time (d) Chinook salmon and steelhead use to pass Lower Granite ladder by ΔT. Estimates were model-averaged means (± se) from annual GLMs for each run. Salmon and steelhead start time was held constant at 12:00 (noon). Base of ladder water temperature was held constant at 10° (open circles), 12° (gray circles), and 15°C (black circles) for spring Chinook salmon, 14° (open circles), 16° (gray circles), and 20°C (black circles) for summer Chinook salmon, 16° (open circles), 18° (gray circles), and 20°C (black circles) for fall Chinook salmon, and 13° (open circles), 17° (gray circles), and 20°C (black circles) for steelhead. These values were the 10^th^, 50^th^, and 90^th^ percentiles of encountered temperatures for each run, rounded to the nearest degree. Note different y-axis scales.

As ΔT increased at Lower Granite Dam, modeled passage times increased for all runs (Figure 5). Predicted mean passage times with ΔT = 0.0°C were 0.2-0.4 d at all modeled temperatures. In contrast, passage times were 1.7-1.8 times longer for spring Chinook salmon when ΔT was 2.0°C. The other runs encountered higher ΔT than spring Chinook salmon, and mean estimated passage times at ΔT = 3.0°C were higher by a factor of 2.0-2.2 (summer Chinook salmon), 4.3-5.3 (fall Chinook salmon), and 2.4-2.5 (steelhead) compared with mean estimates when ΔT = 0.0°C (Figure 5). Patterns were similar at the other three dams. 

### Relationship Between Ladder Temperature and Adult Body Temperature

**Figure 6:**
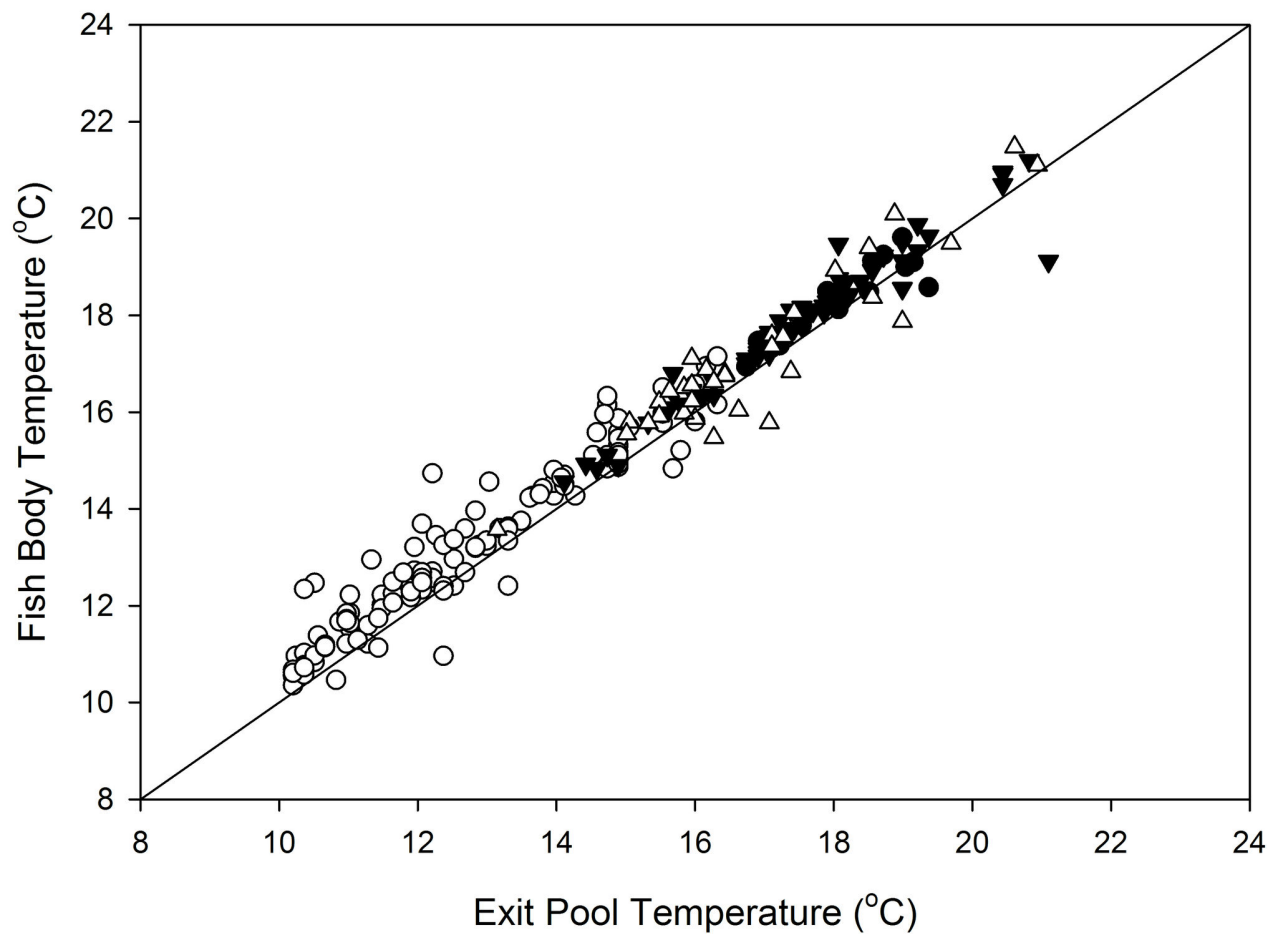
Chinook salmon and steelhead body temperatures in relation to ambient fish ladder water temperatures. Data were collected in 2000 and 2002 at the time fish exited fish ladders (body temperature) and at the top-of-ladder monitoring site at Little Goose Dam (ambient temperatures). Linear regression results were: spring Chinook salmon (○), *n* = 128, *F* = 1378, *P* <0.001, *r* ^2^ = 0.92, slope = 0.86, intercept = 2.71; summer Chinook salmon (Δ), *n* = 34, *F* = 231, *P* <0.001, *r* ^2^ = 0.87, slope = 0.93, intercept = 1.52; fall Chinook salmon (●), *n* = 18, *F* = 155, *P* <0.001, *r* ^2^ = 0.90, slope = 0.95, intercept = 1.14; summer steelhead (▼), *n* = 59, *F* = 856, *P* <0.001, *r* ^2^ = 0.94, slope = 1.00, intercept = 0.94.

Body temperatures of RDST-tagged salmon and steelhead were positively correlated with water temperature inside fish ladders and increased during ladder passage during periods of high ∆T. The relationships exhibited nearly a 1:1 correspondence, demonstrating that most adult body temperatures had equilibrated with the ladder environment by the end of ladder passage events (Figure 6). The close correspondence between fish body temperature and ladder water temperature occurred at all four dams, with r^2^ values ranging from 0.90-0.96 (spring Chinook salmon), 0.88-0.94 (summer Chinook salmon), 0.97-0.98 (fall Chinook salmon), and 0.74-0.97 (summer steelhead). 

The observed rise in body temperature in ladders did not appear to be caused by correlated changes in river temperature outside fishways. For example, there was no evidence of a relationship between ladder ∆T and adult body temperature change during tailrace passage at any dam× run combination (body temperature change in the tailrace, *n* = 10-82; *F* ≤ 3; *P* = 0.10-0.95). Measured body temperatures were consistently ~1°C warmer than surrounding fishway temperatures at the time of exit (Figure 6).

## Discussion

Results indicate that ladder temperature gradients can create a migration obstacle that slows adult salmon and steelhead passage at Snake River dams. While many adults passed during periods of low ∆T, approximately one quarter to one third of the adults in some runs experienced ∆T > 1.0°C. Positive ∆T > 1.0°C was consistently associated with longer radio-tagged fish passage times, confirming our first hypothesis. Fish body temperatures also increased with ∆T during ladder passage, confirming our second hypothesis and suggesting that some adults experienced a departure from their acclimation temperature during dam passage. During the warmest mid-summer river conditions, the additional thermal exposure associated with high ∆T may introduce a heat shock risk [34,35] for adult migrants. This risk may be elevated at Lower Granite Dam, where the frequency and magnitude of positive ∆T was highest. 

The passage delays we recorded were primarily linked to fish exiting the fishways back to the tailrace and then re-entering. Similar behavior was observed in radio-tagged Chinook salmon, sockeye salmon, and steelhead at John Day Dam (lower Columbia River) as ladder water temperatures increased (this study, *unpublished data*). Both cases suggest that adult salmonids perceive a cost to ascending ladders when encountering high ladder ∆T because they slow or reverse upstream movement or search for other routes. 

The general correlation between river temperature and ∆T suggested that river temperature rather than ladder ∆T might explain a portion of the observed behaviors, i.e., that adults were responding to the overall temperature environment and not to ladder temperature gradients *per se*. For example, passage rate through ladders may have slowed at temperatures above the thermal optimum. Clearly, unequivocally separating these effects in an observational study is impossible. However, at least two lines of evidence suggest that mean river temperature was not solely responsible for the slowed migration rates observed at dams. First, ladder ∆T values frequently exceeded 1°C at the 15-18°C river temperatures thought to be optimal for adult swimming [11,32]. Second, examination of tailrace passage times prior to ladder entry in relation to ∆T (results not shown) provided no evidence of spurious correlations. If high river temperatures were correlated with both large ∆T and slowed swimming/dam passage, we would expect to observe both relatively slow tailrace passage and slow ladder passage during large ∆T conditions. Instead, the only significant associations between ∆T and tailrace passage time suggested that swim speeds increased and tailrace passage times decreased during conditions causing higher ∆T (spring Chinook at Little Goose). Operation of the adult trap at Lower Granite Dam may also have affected adult passage behavior at this dam, but the trap was not operated at the warmest ladder temperatures. Trap operation was a potentially confounding factor, but we would expect faster fish passage when the trap was idle, and hence our conclusions at this site were conservative. 

How ladder ∆T affected adult physiology, survival, or reproductive success remains unknown. The body temperature of adults during many passage events at individual Snake River dams occurred at temperatures thought to be physiologically stressful to adult salmonids [12,13]. The additional time fish spent passing dams also may have had adverse effects including increased potential for expression of heat shock protein [34], disease susceptibility [36,37], impaired ovulation [38], increased levels of stress hormone [39], and decreased migration success [24,40]. During the warmest periods, additional passage time and increased temperatures at the top of ladders probably combined to increase potential effects as adults swam near metabolic thresholds, further stressing fish and potentially increasing susceptibility to disease. It remains unknown whether the short-term increases in body temperature (Figure 6) or exposures to high temperature at multiple dams affected subsequent migration success to spawning tributaries, survival during holding and spawning periods or other aspects of the fitness of delayed fish because our monitoring was focused on the impounded system. The potential for cumulative effects of slowed migration and physiological stress seems plausible because nearly all adults entering the Snake River must pass four dams prior to reaching natal tributaries. For example, results of the GLM modeling suggest that an early-run fall Chinook salmon encountering a +1.5°C ∆T at each of the four dams could require several additional days to pass through the four-dam reach, potentially doubling total passage time through the impounded lower Snake River and substantially increasing exposure to stressful temperatures relative to fish that were not delayed. Again, such cumulative delays may contribute to prespawn mortality or other fitness reductions by directly increasing the energetic costs of migration [41], or the indirect effects of additional thermal exposure on disease risk, physiological stress and/or sexual development, all of which may act in a non-linear fashion with rapid increases in mortality beyond threshold values (e.g. 40). We have observed reductions in migration success to spawning tributaries associated with increases in passage time in this system [31], but to what degree slowed passage caused by ladder temperature differences contributes to this pattern is unknown. Currently, migration success through the impounded Federal Columbia River Power System in adult stages is relatively high, but annual estimates do not consistently meet the recommended rates in the Biological Opinion for recovery under the Endangered Species Act. A first step in assessing the physiological effects of hydrosystem passage during high ambient conditions and ladder passage during large ∆T conditions may be to test for elevated heat shock proteins or biomarkers of stress in adults under different temperature exposure levels [42]. 

The cool water releases from Dworshak reservoir are intended to improve flow and thermal conditions for migrating juvenile and adult salmonids. Indeed, available evidence suggests that the releases reduce summer temperatures throughout the lower river, at least below the surface waters [16]. Additionally, adult Chinook salmon [43,44] and summer steelhead [45] appear to select cooler water when available during warm summer conditions. How the local negative effects of Dworshak releases on ladder ∆T at Lower Granite Dam balance against the potential benefits of cooler refugia habitats to migrating adults remains unknown. Although the strongest thermal layering observed was influenced by Dworshak Reservoir releases, layering within other reservoirs resulted from more typical physical limnological processes (solar heating, wind mixing, etc.). Therefore, ladder ∆T may be expected at any gravity-fed passage facility. In fact, ladder ∆T may be greater at dams with larger reservoir storage capacity, longer water residency times, full summer stratification, or characteristics that differ from run-of-river Snake and Columbia River projects (e.g., depth, fetch, transparency, productivity, etc.). 

The local effects of ladder temperature gradients could potentially be alleviated by directing cooler water into the top-of-ladder exit pools. However, modification of water sources and temperature in the forebay just upstream of ladder exits will probably be necessary to ensure that a sharp thermal gradient does not form at the exit and cause migrants to accumulate at the ladder top or retreat downstream in search of more favorable routes. One potential strategy would be to provide a corridor of cool water from the ladder exit through the immediate forebay using suspended water diffusers or some mechanism to create upwelling that would allow adults to exit and sound to deeper, cooler water. Such modifications are currently under consideration at Lower Granite Dam. 

Concerns over fish handling and health prevented radio tagging during the warmest periods of the year. Unfortunately, this constraint resulted in under sampling in the warmer summer months for summer and fall Chinook salmon and steelhead. Consequently, the values presented probably underestimate the true percentages of these runs-at-large that experienced ∆T ≥ 0.5°C. Overall, the under sampling in summer probably led to an underestimation of the true effects of ladder ∆T on the migrating adult populations and was unlikely to have created false associations. Comparisons of the relative effects among dams within run were also unlikely to have been compromised.

The combined effects of the Dworshak Dam cold-water releases, solar heating, and wind setup events collectively explain why ladder ∆T were greatest in frequency and magnitude at Lower Granite Dam. During summer releases from Dworshak Reservoir, ~6° C water released from Dworshak reservoir warms to ~ 10-14°C in the Clearwater River before reaching the confluence with the Snake River where the relatively cool Clearwater River water meets 20-24°C Snake River water. The warmer, lighter Snake River water flows over the Clearwater River input. The thermal stratification created at the confluence persists throughout the Lower Granite Reservoir, with temperature differences of ≥ 5.0°C common between surface and bottom waters through the late summer. Despite considerable vertical mixing at Lower Granite dam, thermal layering again develops in downstream reservoirs. Finer scale circulation patterns in dam forebays also appear to contribute to ladder ∆T, creating differences in ladder ∆T between ladders at individual dams, particularly at Ice Harbor Dam. These differences are likely the result of bathymetry, channel configuration, and circulation patterns in the forebay. 

In conclusion, climate projections for the interior Pacific Northwest are for higher summer temperatures, lower winter snowpack, and consequently, longer, warmer summers with reduced river discharge [46-48]. These projections suggest that management of the Columbia-Snake hydrosystem thermal regime will become increasingly important to the recovery and persistence of Snake River salmon and steelhead, particularly late spring and summer runs (e.g., summer Chinook salmon, sockeye salmon) and early fall-run populations that currently experience the highest water temperatures. Beyond the Snake River, dams worldwide have reservoirs that layer or stratify and potentially create thermal gradients inside downstream fishways. These types of thermal barriers are most likely to disrupt behaviors of cold and cool water migratory species. However, temperate and tropical species could also be at risk when near-surface water temperatures exceed thermal preferences or reach acute levels. In particular, there is potential for a mismatch between cues stimulating migration behavior and the conditions encountered in fish passage facilities. Currently, impoundment-related temperature effects on warm-water species are poorly understood relative to effects on salmonids, but such effects are likely [49,50]. Examination of thermal regimes and related effects on migrant fishes at existing structures may help managers identify modifications that could improve fish passage, whereas consideration of both thermal and hydraulic features should be integral to new fish passage design.
